# Serological, clinical, and risk factors of the Newcastle disease on broilers flocks in Algeria

**DOI:** 10.14202/vetworld.2019.938-944

**Published:** 2019-07-02

**Authors:** Chafik Redha Messaï, Omar Salhi, Djamel khelef, Aziz Lounas, Abdellah Mohamed-Cherif, Rachid Kaidi, Khatima Aït-Oudhia

**Affiliations:** 1 Laboratory of Food Hygiene and Quality Insurance System, High National Veterinary School, Algiers, Algeria; 2 Biotechnology Laboratory of Animal Reproduction, Institute of Veterinary Sciences, Blida, Algeria

**Keywords:** Algeria, biosecurity, enzyme-linked immunosorbent assay, Newcastle disease, serological, vaccination

## Abstract

**Aim:**

The work aimed at studying the serological and clinical factors, as well as the risk factors of the Newcastle disease (ND) on broilers herds in Algeria.

**Materials and Methods:**

A sample of 1248 birds was randomly selected from 52 broiler flocks. We took blood samples from each bird at the level of the wing vein area where an indirect enzyme-linked immunosorbent assay technique was carried out through the use of an IDvet kit.

**Results:**

The flocks showed 82.69% of seroprevalence. Clinically speaking, the most common symptoms were sneezing, rale, greenish diarrhea, torticollis, and motor discords. Most commonly observed postmortem lesions were the proventriculitis, tracheitis, and enteritis. Especially, the caeca are hemorrhagic. The scores show the effect of risk factors. There was a significant effect on the mortality, the hygiene and vaccination groups on antibody titers in time 2. The antibody titers were elevated in the herd that recorded a high mortality (more than 10%) compared with those which recorded a low mortality (<10%) (p=0.002). Therefore, the antibody titers were elevated in herds with bad hygiene, compared with the ones with good hygiene (p=0.04). At last, when broiler chicken were not boosted by ND vaccine, flocks appeared to be more seropositive (p=0.02).

**Conclusion:**

The serological survey conducted in this study provided an important scope for ND as a dominant viral disease in broilers. Many factors are responsible for the onset of these diseases; correct biosecurity measures are needed to reduce the impact of this pathology in poultry farms.

## Introduction

The Newcastle disease (ND) is the most economically important disease in poultry, due to the high rate of morbidity, mortality, slaughter, and associated sanitary measures in poultry farms, particularly in developing countries [1]. ND is caused by virulent strains of avian paramyxovirus type 1. This virus is highly contagious in all the age groups and can infect many species of domestic and wild birds [2]. The major clinical signs of ND are depression, weakness, appetite loss, dehydration, inability to stand, cyanosis of comb, wattle, greenish watery diarrhea, nasal and eye discharges, decreased egg production, and loss of weight followed by death [3]. Gross lesions are petechiae hemorrhages and ulcers with raised borders on the mucosa of the proventriculus, pneumonic lungs, and then hemorrhages in the trachea, air sacs, brain, and spleen [4].

Various diagnostic methods, as the enzyme-linked immunosorbent assay (ELISA), have frequently been used all over the world to detect viruses from the field samples [5,6]. Clinical manifestations and postmortem findings of affected birds may aid to diagnose a disease. Yet, a laboratory diagnosis is necessary for the confirmation of the diseases [7].

Therefore, the present study was undertaken to find out a relationship between the disease diagnostic parameters. Clinical signs, postmortem lesions, and serological tests for the diagnosis of the ND in broilers flocks, to assess the risk factors, were associated with the disease in the affected farms.

## Materials and Methods

### Ethical approval

Experimental procedures were approved by the Institutional Committee for the Protection of Animals of the National Administration of Higher Education and Scientific Research of Algeria (98-11, Act of August 22, 1998).

### Animals

**Figure-1:**
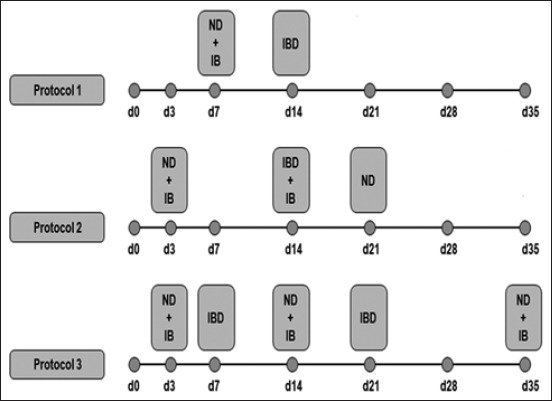
Schematic diagram of protocols vaccine used in the flocks (d: day of vaccine). ND: Newcastle disease, IB: Infectious bronchitis, and IBD: Infectious bursal disease.

The experiment was carried out at commercial farms in the central-east and west of Northern Algeria (longitude 36° and latitude 3°). From July 2016 to December 2017, on 52 broiler flocks with different strains (Arbor acres, Cobb 500, Hubbard F15) aged between 4 and 7 weeks and contained 5000-10,000 birds/farm. The studied flocks were initially vaccinated for ND with live vaccines through different protocols (Figure-1).

For both protocol 1 and 3, the primo-vaccination and booster (recall); the vaccine was bivalent against the Newcastle and infectious bronchitis (IB) diseases. The strains were MA5+ Clone 30. For Infectious Bursal disease, vaccines in the protocol (1), the strain was E228 Intermediate+. In the protocol (3), the strains D78 intermediate was used for primo-vaccination, and for the recall, the strain was E228 Intermediate+.

Concerning protocol 2, the primo-vaccination was bivalent against the Newcastle and the IB diseases. The strains were MA5+ Clone 30; for the booster (recall), it was the strain Villegas–Glisson/University of Georgia (VG/GA) for ND and variant strain IB 4-91 for IB. For infectious bursal disease vaccine strain was E228 Intermediate+. The two strains used against the ND in this study, Clone30 and VG/GA strains were classified as lentogenic.

The analyzed flocks were suspected of acquiring a viral disease (ND) after showing the characteristic, clinical, and necropsy signs.

### Blood collection procedures

A sample of 1,248 birds were randomly selected from 52 broiler flocks (10-15 samples/flock). According to our protocol, two samples were taken from each farm. The first was performed the 1^st^ day after the appearance of the first clinical signs. The second one was done 2-3 weeks time interval, to put in fact the antibodies kinetics in the sera.

Blood samples were collected from the wing vein, in dry tubes and centrifuged (5000 rpm for 10 min) at the same day to recover the sera that were stored in test tubes “Eppendorf,” and frozen at −20°C until analysis.

### Clinical diagnosis

The clinical diagnosis was based on a clinical history from the responsible persons of the farms, including veterinarians in charge of monitoring, recording clinical signs, and gross lesions, which were pathognomonic of the ND on affected chickens through autopsy.

### Serological methods

An indirect ELISA technique was carried out through the use of IDvet Innovative Diagnostics kits (Montpellier, France): ID Screen^®^ NDV Indirect. The sera were diluted to 1/500^th^ and then loaded to ELISA plates to start an immunosorbent reaction as guided by the manufacturer’s manuals. ELx800 spectrophotometer (BioTek™, USA) equipped with the 450-nm filter where the measured optical density was transformed into titrated antibody read ELISA plates. The averages of the titers and the coefficient of variation (CV) were automatically calculated by the band and by series of samples, with the software provided by the laboratory (IDSoft™ 3.9, Montpellier, France).

### Interpretation of the ELISA results

To interpret the ELISA results, the following parameters were taken: The presence of clinical signs and postmortem lesion during the autopsy, the antibodies kinetics; titers between the first and the second sampling. Moreover, mainly according to the Interpreting Poultry Baselines provided by the manufacturer of IDvet ELISA kits: According to the baselines of IDvet, the expected average antibodies titers after the use of one live vaccine, vary from 1000 to 3000 after 3-5 weeks after vaccination. The expected average antibodies titers after the use of two live vaccines, varied from 1000 to 4000 after 3-5 weeks after vaccination. Below the threshold of 1000, it means that there is a poor or no vaccination intake or an immunodepressive disease, and above 3000 for a single live vaccine and 4000 for two live vaccines with a tight CV means that there is a viral passage.

### Observation of the risk factors

A standardized survey was used to assess the risk factors associated with the mortality previously observed. The survey covered the following parameters: Flock characteristics, strain, hygiene, vaccination programs, mortality and morbidity rates, age of occurrence, clinical and necropsy lesions, stocking density, season, area, and climate.

### Statistical analysis

First, descriptive statistics were used to characterize the flocks according to the different factors. Thus, statistical analysis was performed with SAS (Version 9.1.3; SAS Institute Inc., Cary, NC). Before fitting statistical analysis, the examination of the distributions of the antibody titers indicated that using (PROC UNIVARIATE, Shapiro–Wilk test) could not be considered normally distributed. Through time, the antibody titers of the disease were analyzed by fitting the fixed effects of the day. The group and the interaction of the day*group in a repeated measure variance analysis using PROC MIXED models with the random effect of the herd (SAS Inst. Inc. 9.1). Covariance structure used (compound symmetry or autoregressive [AR1]) was chosen and was based on the Akaike information criterion. The layout of our model was as follows: *Y_ijk_* = *µ*+*G_i_*+*T_k_*+*GT_ik_*+*ɛ_ijk_*. Where, *Y_ijk_*=Antibody titer, *μ*=overall mean, *G_i_*=effect of group, *T_k_*=effect of time of sampling (*k*=1 and 2), *GT_ik_*=effect of group × time, and *ɛ_ijk_*=random residual error. Stacked line plots of antibody titer changes were generated using Prism 5.01 (GraphPad Software, Inc. La Jolla, California USA).

## Results

Table-1 presents the scores of antibody titers for ND. Among 52 flocks, 43 (82.69%) that were seropositive, tested to ND. For this mentioned disease, the following has been shown a low CV (CV=31%-44%) and a difference (p<0.0001) in the antibody titer between the first and the second samples (least square of mean [LSM]±standard errors, 1732.06 vs. 4687.00±245.09).

**Table 1:** Serological scores of the Newcastle disease among 52 flocks.

Pathology	Antibody titers		CV(%)	SE	p-value	Seropositivity(%)

	Mean 1	Mean 2				
ND	1732.06	4487.06	31–44	245.1	<0.0001	82.69

In some flocks, we had observed vaccine failure, the titers of the antibodies after the vaccination do not even reach the minimum expected protective threshold. For the remaining 17.31% of the cases, the average titers of the antibodies were in the expected norms of the baselines provided by IDvet; there were no clinical signs or lesions that evoke the ND.

**Figure-2:**
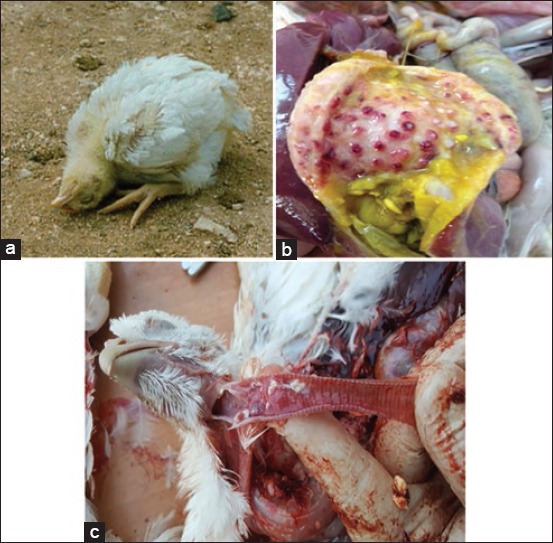
Clinical signs and lesions observed. (a) Torticollis, (b) proventriculitis, (c) tracheitis.

Clinically speaking, the most common signs and lesions of the ND (Figure-2) were respiratory (sneezing and rale), digestive (greenish diarrhea), and nervous (torticollis and motor discords) (Figure-2a). The most commonly observed postmortem lesions were petechiae in the proventriculus (proventriculitis) (Figure-2b), tracheitis (Figure-2c), and enteritis.

Thus, using the necropsy and clinical signs to detect the disease, we observed a very high specificity (85%). In other words, all the birds suspected of having ND had specific antibodies. However, the sensitivities were 75.0%, so for this disease, necropsy, and clinical diagnosis were particularly reliable (Table-2).

**Table 2:** Diagnostic sensitivity (%) and specificity (%), with 95% confidence intervals (CIs) and true prevalence of test based on lesional signs of detecting ND.

Pathology	Sensitivity(%)(95% CI)	Specificity(%)(95% CI)	True prevalence(%)(95% CI)
ND	75.0(69.4, 100)	85.0(100.0, 100.0)	64.5(47.7, 81.4)

CI=Confidence interval

**Figure-3:**
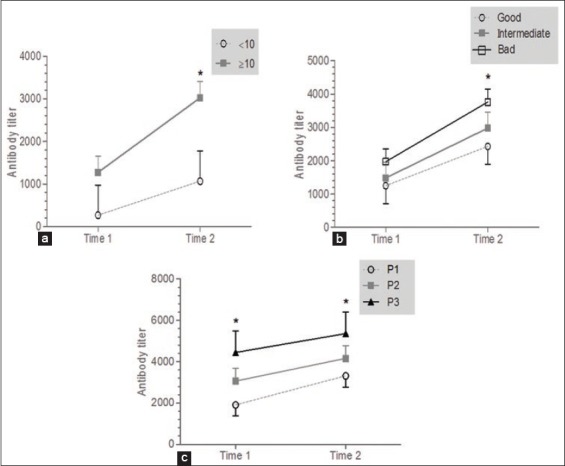
Risk factors affecting ND (a. mortality, b. hygiene, c. protocol of vaccination).

Table-3 shows the effect of risk factors (area, climate, season, age, size of herd, mortality, hygiene, strain, and protocols of vaccination groups) on the amount of antibody titers among time sampling. There was a significant effect of the mortality, hygiene, and protocol of vaccination groups on antibody titers in time 2. The antibody titers were elevated in the herd which recorded a high mortality (>10%) compared with those which recorded a low mortality (<10%) (p=0.002) (Figure 3a).

**Table 3:** Comparison of the least square of means and SEs of antibody titer anti-ND among risk factors (area, climate, season, age, density, mortality, hygiene, strain, and protocols of vaccination groups).

Traits	Group	Time 1	Time 2	SE	p^1^-value	p-value		

						Group	Time	G*T
Area	East	1707.88	3577.88	514.88	0.01	0.25	0.0001	0.88
	Center	1239.89	2659.89	485.43	0.04			
	West	1908.54	3376.08	403.91	0.01			
Climate	Dry	1608.00	3254.67	424.37	0.008	0.98	0.0002	0.85
	Wet	1685.39	3188.61	346.50	0.003			
Season	Autumn	1801.1	3141.50	608.04	0.12	0.82	0.004	0.9
	Summer	1646.25	3325.15	333.04	0.0008			
	Printer	1475.25	2774.75	744.70	0.2227			
Age(day)	≤30	1604.13	3002.25	518.79	0.06	0.67	0.0009	0.79
	>30	1672.73	3292.41	312.84	0.0006			
Density(birds/m^2^)	≤10	1873.50	4022.00	731.21	0.04	0.33	0.0003	0.82
	>10	1777.73	3297.07	377.60	0.006			
Mortality(%)	<10	268.03	1067.36^a^	707.84	0.030	0.002	0.004	0.27
	≥10	1276.18	3027.10^b^	381.79	<0.0001			
Hygiene	Good	1246.57	2427.29^a^	537.56	0.012	0.04	0.0003	0.80
	Intermediate	1481.22	2977.22^ab^	474.09	0.001			
	Bad	1969.71	3761.79^b^	380.12	0.02			
Strain	Arbor Acres	1181.56	2342.56	503.27	0.03	0.16	<.0001	0.47
	Cobb 500	1432.84	1432.84	523.29	0.009			
	Isa	1578.70	3253.89	548.17	0.008			
Vaccination protocol	1	1912.21^a^	3314.65^a^	537.54	0.03	0.02	0.003	0.87
	2	3065.21^ab^	4162.75^ab^	618.19	0.04			
	3	4448.16^b^	5366.03^b^	738.28	0.19			

1 Difference between times for the same group.

a,b Different letters showing a significant difference between groups within the same time sampling. Vaccination protocol, 1-primo vaccine without booster vaccine; 2-primo vaccine with one booster vaccine; 3-primo vaccine with two-booster vaccine. SEs=Standard errors

Therefore, the antibody titers were elevated in the herds with bad hygiene compared with the ones with good hygiene (p=0.04) (Figure 3b). At last, when broiler chicken was not boosted by ND vaccine, flocks appeared to be more seropositive (p=0.02) (Figure 3c). However, there was no significant effect of the climate, season, age, density, strain, and protocol of vaccination groups on the amount of antibody titers among time sampling. There was, on the other hand, a significant effect of sampling time in all the groups. Otherwise, the antibody titers were increased in the sampling time 2.

## Discussion

Our serological scores showed that the farms chosen as samples had a seropositivity rate of 82.7% for ND. For this mentioned disease, a low CV (CV= 31%-44%) has been shown as well as a difference (p<0.0001) in antibody titer between the first and the second samples (LSM±SE, 1,732.06 vs. 4,687.00±245.09). For that reason, an immune status in response to the viral diseases was estimated by measuring the serological response objectified by detection of specific antibodies, previously produced either in response to an infection or following vaccination, on the one hand [8,9]. On the other hand, the protected farms must have a higher average of titers than the protection threshold for all the analysis dates, without being very high compared to the titer resulting from the vaccination. Moreover, this in the absence of specific clinical signs like reported by Abdi *et al*. [10]. Our sample flocks were suspected to be infected with a viral disease, such as ND. They showed typical clinical signs and necropsy signs with high morbidity and mortality. The vaccines used were live vaccines for all the farms.

Clinical and necropsic manifestations of affected birds can help to diagnose a disease, but laboratory diagnosis is needed to confirm it [11]. However, some outbreaks have been reported in the vaccinated populations despite the fact that vaccination is widely applied [12]. Although the ELISA test does not distinguish post-vaccine antibodies from post-infectious antibodies when vaccinated with an inactivated vaccine; the absence or presence of clinical signs and the type of the vaccine should be taken into account [10,13]. For this, we took paired samples; the first sample was taken at the beginning of the disease, while the second sample was taken 2-3 weeks later. In fact, since the concentration of antibodies increases between the two sera collected, this indicates that we had stimulation of the immune system that could be due to a recent infection or to symptomatic viral reactivation [9-11,14,15].

Clinically speaking, the most common signs in our farms were respiratory (sneezing and rale), digestive (greenish diarrhea), nervous signs (torticollis and motor discords), and death. Clinical signs seen on affected farms are similar to the observations of Yune and Abdela [11], Banerjee *et al*. [16], and Brown and Bevins [17]. The most commonly observed postmortem lesions in our farms were petechiae in the proventriculus or proventriculitis, tracheitis, and enteritis. These findings match with the observations of Yune and Abdela [11], De Oliveira Torres Carrasco *et al*. [18], Brown and Bevins [17], Brar *et al*. [19], Crespo *et al*. [20], and Talha *et al*. [21].

As far as factors affecting ND are concerned, there was a significant effect of mortality, hygiene, and protocol of vaccination groups on antibody titers in time 2. The antibody titers were elevated in the flock that recorded high mortality (>10%) compared with those which recorded a low mortality (<10%) (p=0.002). Therefore, the antibody titers were elevated in the flocks with bad hygiene compared with those with good hygiene (p=0.04). At last, when broiler chicken was not boosted by ND vaccine, flocks appeared to be more seropositive (p=0.02). Regarding mortality, ND has been a very serious problem for poultry production in many countries. The disease causes high economic losses due to the high rate of morbidity and mortality [22]. The ND caused by mesogenic strains may cause mortality that can reach 25%, whereas those which are velogenic strains, may reach up to 100% and can even vary from 80% to 90% as far as adults are concerned [8,11,23,24]. It is clear that good hygiene and biosecurity measures aim at preventing the introduction of viruses into poultry farms, thus, reducing their economic losses [14,25-27]. Therefore, in response to the threat presented by ND, several countries have put in fact vaccination campaigns to prevent epizootics. However, outbreaks have been reported in vaccinated populations despite the fact that vaccination is widely applied [28,29]. It is known that the vaccination of poultry provides an excellent means to lessen the clinical signs of infection caused by virulent ND virus (NDV) [29-31]. It has also been known, for a long time, that the vaccination itself (with live vaccines based on non-virulent virus strains) may cause disease and reduced growth among the vaccinated birds [29,30]. Alsahami *et al*. [9], Mayers *et al*. [30], and Orajaka *et al*. [32] had reported that the vaccination is the only safe option when it comes to the control of the infection strategies.

Incidence of ND in vaccinated flocks may be due to inadequate vaccination practices or biosecurity flaws, as signaled by Dortmans *et al*. [26]. In our study, the entire 52 flocks were vaccinated by live vaccine strain through drinking water. We had observed vaccine failure, which may be due to the following factors: Inadequate vaccination method; to put it differently, the bad water quality, water that may contain disinfectants, which neutralizes the live vaccine. The insufficient number of troughs in the farms, the non-respect of the cooling chain of the vaccines storage, and the non-use of the vaccine stabilizers during its preparation in water, are causes of vaccine failure. Moreover, probably to the immuno-suppressive diseases that reduce the immune response, such as the infectious bursal disease, reovirus, the infectious anemia, and mycotoxins. In addition, the administration mode through the oral route through drinking water is not the recommended technique for viruses with a respiratory tropism. The nebulization would have given better results and more protection for poultry.

Alsahami *et al*. [9] and Bulbule *et al*. [33] reported that the NDV vaccine of different strains was being used to control clinical disease during the outbreak. In addition, control of risk factors, including immunosuppressive agents, biosecurity breaks, inadequate management practices, and harsh environment together is required to diminish the economic impact of ND outbreaks. However, Markos and Abdela [34] reported that the principal management procedures should include strict biosecurity measures, which help in preventing the spread of infective material from house to house and from farm to farm. In fact, Yune and Abdela [11] had reported that good biosecurity could protect the poultry flocks from the ND.

## Conclusion

This serological study conducted in this assay had provided an important scope about ND as a dominant viral disease on the broiler chickens and had found that the majority of the broiler flocks were seropositive. Clinical manifestations and postmortem findings of the affected birds may assist in diagnosing a disease. However, a laboratory diagnosis is necessary for the confirmation of the disease. However, the findings also suggest that the risk factors related to poor biosecurity measures, inadequate vaccination program, and farm practices appear to have a significant role in the severity of the disease already observed in the affected farms. If those factors were alleviated, the severity of the ND problems in farms would greatly reduce. Further investigations are recommended to identify the circulating virus genotypes and models of transmission for a better understanding of ND epidemiology in the broilers flocks in Algeria.

## Authors’ Contributions

CRM, OS, DK, AL, AM, RK, and KA designed the study. CRM and OS wrote the manuscript and collected data. OS and AL diagnosed the disease and collected the samples. CRM, OS, and AM carried out the laboratory work. OS performed statistical analysis. CRM, OS, DK, and AL analyzed the data. CRM, OS, and AL reviewed the manuscript. All authors read and approved the final manuscript.
